# “Follow the Whistle: Physical Activity Is Calling You”: Evaluation of Implementation and Impact of a Portuguese Nationwide Mass Media Campaign to Promote Physical Activity

**DOI:** 10.3390/ijerph17218062

**Published:** 2020-11-02

**Authors:** Marlene Nunes Silva, Cristina Godinho, Marta Salavisa, Katherine Owen, Rute Santos, Catarina Santos Silva, Romeu Mendes, Pedro J. Teixeira, Graça Freitas, Adrian Bauman

**Affiliations:** 1 Programa Nacional para a Promoção da Atividade Física, Direção-Geral da Saúde, 1049 Lisbon, Portugal; cristinagodinho@dgs.min-saude.pt (C.G.); rsantos.ciafel@fade.up.pt (R.S.); catarinasantosilva@dgs.min-saude.pt (C.S.S.); romeuduartemendes@gmail.com (R.M.); pteixeira@fmh.ulisboa.pt (P.J.T.); 2 Faculdade de Educação Física e Desporto, CIDEFES, Universidade Lusófona de Humanidades e Tecnologias, 1749 Lisbon, Portugal; 3 CIPER, Faculdade de Motricidade Humana, Universidade de Lisboa, 1495 Cruz Quebrada Dafundo, Portugal; 4 Católica Research Centre for Psychological—Family and Social Wellbeing, Universidade Católica Portuguesa, 1649 Lisbon, Portugal; 5 NOVA National School of Public Health, Universidade NOVA de Lisboa, 1099 Lisbon, Portugal; marta.salavisa@gmail.com; 6 School of Public Health and Charles Perkins Centre, The University of Sydney, Camperdown, NSW 2050, Australia; katherine.owen@sydney.edu.au (K.O.); adrian.bauman@sydney.edu.au (A.B.); 7 Research Centre in Physical Activity and Health, Faculty of Sport, University of Porto, 4200 Porto, Portugal; 8 EPIUnit—Instituto de Saúde Pública, Universidade do Porto, 4050 Porto, Portugal; 9 Direção-Geral da Saúde, 1049 Lisbon, Portugal; gracafreitas@dgs.min-saude.pt

**Keywords:** physical activity, healthy lifestyles, mass media campaign, evaluation, social marketing

## Abstract

To raise perceived capability (C), opportunity (O) and motivation (M) for physical activity (PA) behaviour (B) among adults, the Portuguese Directorate-General of Health developed a mass media campaign named “Follow the Whistle”, based on behaviour change theory and social marketing principles. Comprehensive formative and process evaluation suggests this media-led campaign used best-practice principles. The campaign adopted a population-wide approach, had clear behavioural goals, and clear multi-strategy implementation. We assessed campaign awareness and initial impact using pre (*n* = 878, 57% women) and post-campaign (*n* = 1319, 58% women) independent adult population samples via an online questionnaire, comprising socio-demographic factors, campaign awareness and recall, and psychosocial and behavioural measures linked to the COM-B model. PA was assessed with IPAQ and the Activity Choice Index. The post-campaign recall was typical of levels following national campaigns (24%). Post-campaign measures were higher for key theory-based targets (all *p* < 0.05), namely self-efficacy, perceived opportunities to be more active and intrinsic motivation. The impact on social norms and self-efficacy was moderated by campaign awareness. Concerning PA, effects were found for vigorous activity (*p* < 0.01), but not for incidental activity. Overall the campaign impacted key theory-based intermediate outcomes, but did not influence incidental activity, which highlights the need for sustained and repeated campaign efforts.

## 1. Introduction

Data from the 2017 Eurobarometer survey place Portugal as one of the four European Union countries with the highest rates of physical inactivity, with 79% of the Portuguese adults referring that they “never or seldom” engage in structured physical activities or sports, and 74% indicating “never or seldom” engage in other forms of physical activity [1].

The most frequently reported barriers to physical activity in Portugal are the lack of motivation and interest (33%), as well as the lack of time (26%), or it being too expensive (19%) [1]. Moreover, data from a survey with a representative sample of the adult population revealed that more than a half of the population (58%) did not recognize daily activities such as climbing stairs as forms of physical activity, and only 2% were aware of World Health Organization (WHO) physical activity recommendations [2].

One of the proposed strategies to promote physical activity is the use of large scale mass media campaigns [3]. The Global Action Plan on Physical Activity (GAPPA) strategy of the WHO recommends the creation of active societies, starting with influencing social norms towards inactivity through best-practice community-wide communication campaigns. Examples of successful population-level physical activity campaigns have been reported for over 30 years [4]. The initial purposes of physical activity campaigns are to increase awareness and recall of the message, and influencing intermediate outcomes, such as attitudes towards physical activity and self-efficacy [5,6]. Strategies include mass reach communications, social media, and following the principles of social marketing, which includes supportive marketing, community events and environmental change components [7]. Such campaigns need to be evaluated using clear formative (pre-campaign) research and careful process monitoring to assess implementation [8]. In doing this, a sound conceptual framework may be helpful, including using insights from social marketing and socio-ecologic approaches. Systematic reviews pointed out the ongoing lack of use or underreporting of the use of theory in social marketing campaigns, reinforcing the call for applying theory to guide and evaluate interventions [9,10].

A mass media campaign was developed in Portugal, following the global launch of the GAPPA in June 2018, in Lisbon. Consistent with the GAPPA/WHO recommendations, the national campaign focused on developing innovative and relevant messages, in order to increase awareness that physical activity includes different types of incidental daily activity behaviours, including climbing stairs, active commuting and recreational activities, such as play and dance. In 2019, the mass media campaign “Siga o Assobio/Follow the Whistle: Physical activity is calling you” was implemented by the Portuguese National Programme for Physical Activity of the Directorate-General of Health (DGS). This campaign targeted 35 to 60 years old adults and was based on social marketing principles applied to health promotion [11] and the COM-B model [12].

The COM-B model proposes that behaviour change requires (i) Capability, which pertains to the physical and psychological ability to engage in physical activity; (ii) Opportunity, which includes all forms of physical and social opportunities for activity; and (iii) Motivation, both stemming from reflective and automatic psychological processes stimulating the initiation and maintenance of physical activity. As mentioned, the campaign “Follow the Whistle” was also built on a social marketing approach [11], in which encouragement was used to make active choices seem more favorable, and messages were embedded within a cultural context.

In the development of the campaign and its central messages, formative research comprised pre-production qualitative research with the target population (through six semi-structured focus groups). The formative research identified key beliefs about physical activity and contributed to the development of central campaign themes and messages [13,14].

A detailed description of the formative research is reported elsewhere [15]. Briefly, six focus groups with 36 adults (aged 30+ years) were conducted in order to identify and explore key messages and strategies when developing a physical activity promotion campaign specially targeted to individuals with sedentary lifestyles; from these, it was possible to identify the need to reframe the traditional understanding of what “physical activity” is, by highlighting the importance of non-structured physical activities, and the ease of integrating these into daily-life, saving time and increasing enjoyment. The value of physical activity as intrinsically motivating was also identified. Results from the focus groups matched identified epidemiological concerns, namely that more than half of the population did not recognize incidental activities such as climbing stairs as physical activity, and reported time, cost and motivational issues as barriers to physical activity participation. This meant that the campaign messages should highlight pleasurable and easy-to-perform activities that may also potentiate social interaction and are compatible with other important valued activities of daily life.

Based on these findings, and the COM-B model, the contents from “Siga o Assobio/Follow the Whistle” media campaign (available here) small stories—one for each of the six characters—were also produced for television (TV) on the three main Portuguese channels) aimed to change motivation (e.g., showing different characters performing fun and meaningful physical activities alone or with special others, and that no extra time, money or special equipment are necessary). The message also portrayed opportunities to be more physically active (e.g., active play with children, walking instead of waiting for the bus, climbing stairs instead of taking the lift, going to work cycling) and fostered capability (e.g., even if one is not in shape or has a physical disability, there are physical activities suited for every level of fitness).

Data-based methods for audience segmentation were also used to identify the campaign target group. Social marketing mix components like product benefits (physical activity is easy, positive and time-efficient), reducing barriers (lack of time or money to train) and competition (stairs vs. lift, bike vs. car) were applied, as well as place (distribution of campaign flyers on public transportation, social media advertisements (Facebook and Youtube), DGS and Ministry of Health websites) and promotion (persuasive communications). Formative research also led to important marketing strategy decisions, such as street roller skating excluded from the campaign, because the target age group did not recognize it as an activity that the people usually do in Portugal.

The aim of this paper is to describe the implementation and initial impact of the effectiveness of the mass media campaign “Siga o Assobio/Follow the Whistle”.

## 2. Materials and Methods

### 2.1. Campaign Implementation Description

**Figure 1:**
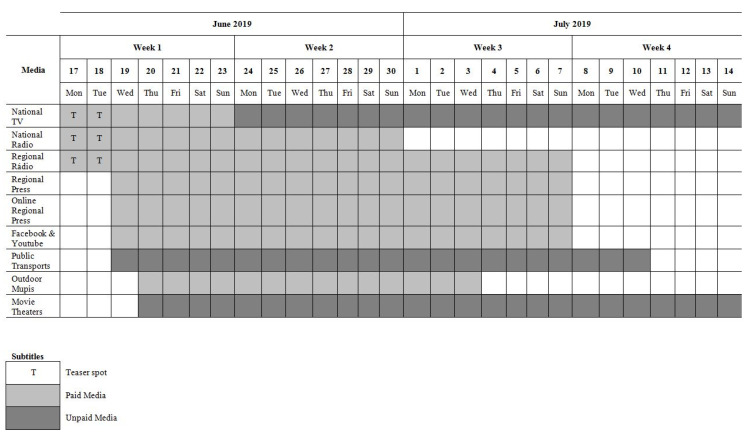
Campaign schedule.

The “Follow the Whistle” mass media campaign was launched on June 16th, 2019 and continued for four consecutive weeks. The campaign started with a five-second teaser media message broadcasted on the TV and the radio for two days. Then a 20 s “revelation message” was broadcast on TV, radio, online and in cinema for five days. Finally, a mix of the five and 10 s reminders was broadcast across media channels for 13 days. The campaign messages were also aired on national and regional radio, and shown in movie theatres, outdoor advertising, and on public transport, as well as print media in regional publications. Figure 1 provides the complete campaign schedule.

### 2.2. Study Design and Procedures

#### 2.2.1. Process Evaluation

Comprehensive process evaluation describes campaign implementation, acceptance and population reach [8]. This monitors implementation fidelity, target population reach and exposure, and assesses activities of the campaign and media news coverage.

Table 1 (see results section) provides a short description of the dimensions that were evaluated (i.e., Implementation fidelity reflecting the extent to which the campaign was carried out as planned; Reach and exposure to the Targeted Audience) and results achieved.

#### 2.2.2. Impact Evaluation

##### Study Participants

Pre-post independent population samples were used to assess the campaign impact. The two surveys used the Qualtrics platform and were distributed through the mailing list of a Portuguese mutualist insurance company (“Associação Mutualista Montepio”). The pre-campaign survey was online between 3 and 13 June 2019 and the post-campaign survey was conducted from 17 July to 1 August 2019. As mentioned above, participants were recruited via the “Associação Mutualista Montepio” members’ network, comprising of 625,419 members from all regions of Portugal. From these, a panel of 20,000 adults agreed to participate, and from these around 10% participated (see results for descriptive details of participants).

The study goal was explained in the email, prior to the beginning of each survey, and data were confidentiality assured. Members agreeing to participate provided informed consent in compliance with the Declaration of Helsinki, and with all European Union relevant directives and the related Portuguese legislation on ethics involving research in humans and personal data processing.

### 2.3. Measures

An online self-administered questionnaire was comprised of socio-demographic factors, psychosocial and behavioural measures, as well as measures on campaign awareness and recall. Following the four categories of the COM-B model, both questionnaires included items to assess perceived capability for physical activity (C), perceived opportunity for practice/ease of integration of physical activity in daily living (O), motivation for physical activity practice (M) and behaviour (B). For all items (except for measures of behaviour) a Likert-type response scale was used, ranging from 1 (totally disagree) to 7 (totally agree).

#### 2.3.1. Campaign Awareness and Recall Measures

The pre-campaign questionnaire included 3 awareness items: i. “In the past four weeks, have you seen any message about physical activity in the media?” (Yes/No); ii. (if yes) “What do you recall about these ads? Please, briefly describe specific aspects you remember.” (short open-ended answer); and iii. “Have you seen the message “Follow the Whistle”?” (Yes/No). This last item was included to identify potential “ghost campaign” recall.

In addition, the post-campaign questionnaire asked those who had seen the “follow the whistle” message question regarding i. “What was the main message of the campaign?” (open-ended answer); ii. “Where did you see, read or hear any part of this/these advertising or messages? (multiple closed-coded response options).

In order to collect information about specific campaign message recognition, 14 randomly shown images (7 from the “follow the whistle” campaign, 7 from other campaigns) were presented and participants were asked to choose the images they remember seeing in the previous month. For those that remembered at least one specific “follow the whistle” campaign image, additional questions were asked about campaign appreciation, message understanding and message salience, asked respectively: i.,” to what extent did you like or not these ads?”; ii. “In your opinion, what was the main message of the “follow the whistle” campaign?”; and iii. “rate your level of agreement with the following sentences, where 1 means “nothing” and 7 means “a lot”: (a) Did you find the message useful? (b) Is the message personally relevant for you?”.

#### 2.3.2. COM-B Indicators

Perceived capability (C) was assessed using a pool of proximal indicators related to:

- Knowledge, adapted from the Portuguese Physical Activity Barometer [2] and the Eurobarometer on Sports and Physical Activity [1]: “Climbing stairs or walking are not physical activities”, “Physical activity has to be vigorous to be useful”, and “Practicing regular physical activity improves my quality of life”.
- Perceived self-efficacy, adapted from Bandura (2006) [16]: The stem “I am confident that I can keep active on a regular basis, even if…(limited time, not in shape, no specific equipment).” Another item assessed self-efficacy beliefs regarding physical activity practice in daily life [2]: “Currently, it is relatively easy for me to walk and bicycle for at least part of my daily journeys”. The five items had good internal consistency (Cronbach alpha = 0.76), and were summed to a PC score.

Perceived opportunity for practice (O) was assessed using items adapted from the Eurobarometer on Sports and Physical Activity [1]: “I can integrate physical activity into my everyday life”; “There are no opportunities to be active in the area where I live” (reverse coded); “Even for those who want, there are not many free options for being active (reverse coded)”; “In my day-to-day life, there are spaces, situations or people that encourage me to move more”. The perceived opportunity items showed modest internal consistency (Cronbach alpha = 0.50) and were not summed.

Motivation (M) for physical activity was assessed via a pool of proximal indicators related to:

- Interest and enjoyment (2 items adapted from Exercise Regulations Questionnaire (BREQ-3) [17]: ”Physical activity is as important as other things in my life”, “I enjoy practicing physical activity”);
- Internal Locus of Control [18] (“I do physical activity because I want to, not because I have to”);
- Positive Attitude (Two items based on Norman, Conner and Bell (2000) [19] were used to assess positive attitudes towards physical activity. The stem “I see physical activity as something that is…” was followed by (i) “Enjoyable”; (ii) “Interesting”. (3-item motivation, Cronbach alpha = 0.82, summed as a score));
- Intention to increase physical activity practice (assed using the item “I intend to increase my physical activity in the near future”, adapted from Sparks et al. (2004) [20]);
- Social norms were assessed through the following items:
  ○ “Where I work/live, I often see other people being active”
  ○ “I know a lot of people who are not active” (reverse coded)
  ○ “There are more people who use public transport or bicycles compared to cars”
  ○ “There are more and more people being active” (4-item poor Cronbach alpha = 0.27, items analyzed individually).

Physical Activity Behaviour (B) was assessed via self-report, using the International Physical Activity Questionnaire (IPAQ)—short version [21] to estimate the total level of physical activity (categorized as low, moderate, high). Lifestyle physical activities integrated in daily activities were assessed using the Activity Choice Index questionnaire [22] (e.g., using stairs instead of the lift; walking instead of using transportation; choosing to stand up instead of sitting).

### 2.4. Data Analysis

Frequencies and proportions for demographic characteristics and health conditions were calculated for pre-campaign and post-campaign respondents. Chi-squared tests were used to determine whether there were significant differences between the respondents surveyed pre-campaign and post-campaign.

Generalized linear models were conducted to examine differences in intermediate variables (i.e., perceived capability, perceived opportunity, motivation, physical activity literacy, social norms, and physical activity intentions) and physical activity levels between pre-campaign and post-campaign respondents. We examined differences in intermediate variables and physical activity levels between respondents who were aware of the campaign and respondents who were not (Supplementary Tables). These models were adjusted for age, sex, education, depression, anxiety, nil diseases and other diseases. We also calculated Cohen’s *d* for mean differences and Cohen’s *h* for proportion differences, which were interpreted as 0.2 for small, 0.5 for medium and 0.8 for large [23]. All analyses were performed in SAS Enterprise Guide 9.4 (SAS Institute, Cary, NC, USA).

## 3. Results

### 3.1. Process Evaluation

Table 1 summarizes the process evaluation dimensions and results.

### 3.2. Impact Evaluation

The samples pre-and post-campaign are shown in Table 2 and were broadly demographically similar. Data were confined to the target age group of 30–65 years. Those in the post-campaign survey were somewhat younger, slightly more with higher education, and showed slightly better self-rated health, but these differences were generally small.

**Figure 2:**
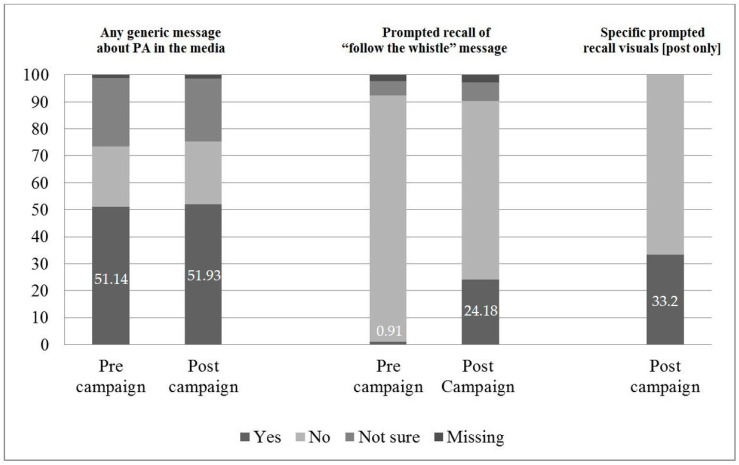
Campaign recall (%) (generic, text prompted, and visuals prompted).

Figure 2 shows changes in campaign recall. There was no change for “any generic physical activity message” recalled in the media, but a significant increase was seen from 1% to 24% in prompted campaign tagline recall, and one third at post-campaign correctly recalled specific campaign visual messages.

Table 3 shows COM-B proximal indicators. Post-campaign values were significantly higher for a number of these indicators, namely self-efficacy (with the exception of one item), perceived opportunities to be more active and several items tapping on intrinsic motivation (enjoyment, interest, internal locus of causality). Effect sizes were overall of statistically small magnitude.

High physical activity was reported by 54.4% (95%CI: 51.1–57.7) at the baseline, which was significantly higher at post-campaign—64.5% (95% CI: 61.9–67.1, *p* < 0.001). Mean physical activity MET minutes are shown in Table 4, with a significant post-campaign increase only for the vigorous activities’ category. Unadjusted data were similar for physical activity MET-minutes (Supplementary Table S1).

Table 5 shows changes in the likelihood of reporting incidental physical activities “often”. Although there were small increases in the proportions using the stairs and walking, these (and other incidental behaviour questions) were not significantly different at post-campaign, compared to pre-campaign levels.

Post campaign analyses were conducted to examine the relationship between awareness of the campaign and outcome variables. Those aware of the campaign, using prompted or specific campaign image recall, showed similar levels of physical activity (Supplementary Table S2). Additionally, incidental lifestyle physical activity behaviours reported “often” did not vary by campaign awareness (Supplementary Table S3).

Several of the COM-B proximal indicators, including physical activity beliefs about planning, integrating into daily life and opportunities to be active, as well as self-efficacy items were significantly higher among those aware of the campaign; also, important markers of intrinsic motivation (i.e., enjoyment, interest) were higher among those who recognised the campaign compared to those unaware of it (Supplementary Tables S4 and S5).

## 4. Discussion

Evaluations of national physical activity campaigns are infrequently reported from Europe [4,24], nonetheless, overall evidence has pointed out that the most effective health communication campaigns are informed by formative research and engage in comprehensive communication strategies based on an understanding of behavioural determinants and using social media and interpersonal communication as part of the strategy. Furthermore, while many campaigns have failed to invest in evaluation, there is a growing recognition of the importance of rigorous outcome evaluation [25]. In this regard, evidence claims that campaigns should focus more on influencing proximal variables, theoretically based, to bring about long-term behaviour change. Evaluation designs that measure the full range of proximal and intermediate variables are preferred to a focus on behaviour change alone [26]. Furthermore, consistent evidence supports the importance of: (i) physical activity messages to be framed positively and highlighting social and mental health short-term outcomes; (ii) message content be targeted to specific audiences; (iii) formative research, psychological theory and/or social marketing principles for developing physical activity messages [27].

In alignment with these recommendations, the “follow the whistle” campaign targeted sedentary urban adults and depicted physical activity as something positive, that adds to life and that allows sharing with other important ones, promoting a closer connection with the physical and social environment. Comprehensive process evaluation metrics (Table 2) show the substantial investment in the campaign, with high reach in mainstream media, and reasonably high engagement through social media. The main messages implicit in the stories of “follow the whistle” campaign characters were to highlight the short-term social (e.g., physical activity as an opportunity to connect with others) and mental (e.g., improved mood and energy levels) health benefits. This recommended focus comes from literature targeting some of the main outcomes as in “follow the whistle”: motivation and self-efficacy [28,29,30].

By using the COM-B model, this Portuguese campaign made use of psychological theory to identify behavioural determinants and to tailor physical activity messages: (C) physical activity is easy to perform (it can be adapted to all sorts of physical condition); (O) several opportunities to be physically active exist daily, at no extra economic cost; (M) being active is fun, compatible with other valuable activities in life.

The comparison of pre and post-campaign measures identifies significant positive intermediate campaign effects, specifically:

- Increased awareness of the campaign and understanding the campaign message
- Perceived self-efficacy for physical activity practice, even when one can “have little time”, “not being in the best physical shape” or “not having specific equipment”;
- Perceived opportunity to practice physical activity, such as “integrating physical activity into daily life” and as having “opportunities to be active in the area of residence”;
- Motivation for physical activity, considering that physical activity is pleasant, interesting and compatible with other life goals, driven by an internal locus of causality, important markers of intrinsic motivation.

It is important to note that although, in statistical terms, the magnitude of the effects on these indicators may be considered small [23], even small effects in a risk factor among a large group of people can lead to meaningful changes at the population level [31]. Furthermore, small effects are expected from mass media campaigns alone, and following a hierarchy-of-effects model, most of the effect is observed in awareness and understanding of the campaign issue. Post-campaign results here show prompted and unprompted awareness rates and are typical of population-wide physical activity mass media campaigns [4,24]. Social norms and efficacy were also higher amongst those aware of the campaign (despite not directly impacted).

A meta-analyses [32] on the effects of health campaigns (overall, not physical activity-specific) reported that for adults, the average random effect size was r = 0.09, indicating a small effect. Specifically for physical activity campaigns, a recent scoping review [27], as well as numerous evaluations of single mass media campaigns [4,24,33,34,35,36,37], reported mixed findings on proximal outcomes (e.g., awareness and campaign recall) and intermediate outcomes (e.g., intention to be active); but, generally campaigns had less effect on intermediate outcomes than on proximal, with even more mixed effects on distal outcomes such as physical activity behaviour. This reasoning explains the modest, but encouraging effects of the “Follow the Whistle”, relative to previous physical activity campaigns.

Indeed, no changes were found in incidental activity, despite the fact that campaign images and messages were related to those types of physical activities. The literature clearly identifies that sustained campaign efforts (e.g., over time with serial campaigns) are usually needed for behaviour changes to occur. This may be especially applicable to lifestyle physical activity, which is dependent on several other determinants such as habit and the built environment, as well as the availability of community-based programmes, resources and policies that support practice [38].

An additional explanation for the lack of effects on incidental physical activity (vs. post-programme significant effect on vigorous activity), should be explored in further studies, and should concern the critical (and under-researched) issue of the needed amount or intensity of instrumental behaviours that allow people to notice a behaviour change (intensity is notable). A recent paper [39] has shown that the intensity of physical activity may be crucial to people’s perception of change. People may misconstrue lower-intensity lifestyle physical activity (not noticing or valuing changes). Further research could include more objective device-based measures of physical activity to overcome the possible biases of individual perceptions.

In sum, the campaign had clear strengths, namely a sound theory-based design, planned using formative evaluation, and a clear innovative concept and image. There were some weaknesses, with no effects on targets such as daily physical activities: physical activity measures were self-reported, the panel of respondents (pre/post-campaign), although almost identical, were not the same. Furthermore, the observational nature of the study and the short period assessed may preclude detecting later changes. Indeed, populational changes in walking and in incidental physical activity are likely to require more sustained campaign efforts over time to be successfully adopted.

## 5. Conclusions

Portugal has one of the highest rates of physical inactivity in Europe. Perceived capability, opportunity and motivation for physical activity are important determinants of physical activity among adults, according to the COM-B model of behavioural change. Thus, based on that framework and also on social marketing principles, a national mass media campaign (“Siga o Assobio/Follow the Whistle”) was implemented (e.g., TV, radio, online, cinema, newspapers, and outdoors) and tested by the National Programme for Physical Activity Promotion of the Portuguese Directorate-General of Health. Immediate post-campaign results are encouraging, based on the formative, process and proximal impact evaluation findings. Initial campaign effects on proximal outcomes (e.g., awareness) followed by intermediate (e.g., motivation) were realised, but sustained ongoing population strategies may be required for population behaviour change [4,24,27,32]. Making changes to lifestyle habits and influencing incidental activities remain to be achieved, and subsequent waves of this national campaign are needed to reinforce incidental or automatic physical activities. Reactivation of the campaign (planned for the spring of 2020 but postponed due to the COVID-19 pandemic) will allow further resolution of assessing the campaign’s impact.

## Figures and Tables

**Table 1:** Process evaluation dimensions and results.

Process (Implementation) Evaluation Dimensions	Indicators		Results
Implementation fidelity (The extent to which the campaign was carried out as planned)	Campaign calendar schedule		Campaign started on the scheduled date and lasted longer than expected on Portuguese Football Federation (FPF) cable channel.
	Stakeholders Engagement	Partnerships	Partnership engagement level was enough for the proper execution of the campaign. The campaign had 3 types of partnerships: (1) campaign partners: Portuguese Institute of Sports and Youth (IPDJ) and FPF; (2) dissemination partners: transport, cinemas, TV channels; (3) process evaluation partners: “Associação Mutualista Montepio” and Lisbon Municipality
		Event attendance	All campaign’s target stakeholders (from health, education, transport, sport, municipalities sectors) were present.
	Media Coverage	Campaign launch	20 news
		Campaign event	5 news
	Campaign storyline as planned		The campaign ran as planned.
	Campaign Website		Trafic: 2326 visits
Reach and exposure to the Targeted Audience	TV		Number of spots: 212; GRP: 998; Reach on target audience: 88% (33,553,960); OTS: 12.5
	Radio	National	Number of stations: 2; Number of spots: 316
		Regional	Number of stations: 20; Number of spots: 5880
	Internet	Facebook	Number of clicks: 11,645; Number of impressions: 172,953; CTR: 6.7; Interactions: 8138—reactions: 1685|Shares: 544|Comments: 18
		Youtube	Number of views: 130,940; Impressions: 346,204; VTR:37.8
		Online Regional Media	Number of media: 19; Views: 650; Clicks: 600
	Outdoor		500 mupis
	Print Regional Press		Number of media: 19; Number of prints: 38

**Table 2:** Demographic characteristics for all respondents pre- and post-campaign.

	Pre-Campaign		Post-Campaign		
	*n*	%	*n*	%	*p*-Value
**All persons**	878	100	1319	100	
**Age category**					<0.001
30–39	134	15.26	339	25.70	
40–49	353	40.21	510	38.67	
50–59	247	28.13	303	22.97	
60–65	144	16.40	167	12.66	
**Sex**					0.52
Male	379	43.17	551	41.77	
Female	499	56.83	768	58.23	
**Education**					<0.01
High school or less	300	34.17	371	28.13	
Above high school	564	64.24	922	69.90	
Missing	14	1.59	26	1.97	
**Employment**					0.49
Employed	713	81.21	1096	83.09	
Other	157	17.88	214	16.22	
Missing	8	0.91	9	0.68	
**Area of residence**					0.37
Central Urban	405	46.13	649	49.20	
Other	465	52.96	659	49.96	
Missing	8	0.91	11	0.83	
**Global self-rated health**					<0.001
Bad	324	36.90	389	29.49	
Good	542	61.73	916	69.45	
Missing	12	1.37	14	1.06	
**Health conditions**					
Diabetes	26	2.96	47	3.56	0.44
Cardiovascular Diseases	13	1.48	26	1.97	0.39
Hypertension	132	15.03	170	12.89	0.15
Depression	76	8.66	63	4.78	<0.001
Anxiety	119	13.55	138	10.46	0.03
No diseases	461	52.51	779	59.06	<0.01
Other diseases	192	21.87	241	18.27	0.04

**Table 3:** Adjusted COM-B proximal indicators for all respondents’ pre-campaign and post-campaign (increasing scores indicate increased agreement).

	Pre-Campaign M (SE)	Post-Campaign M (SE)	Effect Size (Cohen’s *d*)	*p*-Value
***Perceived capability***				
**Knowledge**				
Climbing stairs or walking are not physical activity	2.35 (0.07)	2.17 (0.06)	−0.09	0.05
Only high intensity activity has benefits	1.61 (0.04)	1.57 (0.04)	−0.03	0.44
**Self-efficacy**				
I am confident that I can keep active on a regular basis, even when...				
I have little time	4.24 (0.06)	4.65 (0.05)	0.25	<0.001
I’m not in great physical shape	4.49 (0.06)	4.88 (0.05)	0.24	<0.001
I do not have specific equipment	4.75 (0.07)	5.07 (0.06)	0.17	<0.001
I do not have much money	4.92 (0.07)	5.07 (0.06)	0.08	0.09
I can integrate physical activity into my day	4.83 (0.06)	5.45 (0.05)	0.38	<0.001
Currently, it is relatively easy for me to walk or bicycle for at least part of my daily journeys	4.06 (0.08)	4.33 (0.06)	0.13	<0.01
***Perceived opportunity***				
**Physical and social environment**				
There are many opportunities to be active where I live	5.20 (0.06)	5.39 (0.05)	0.12	<0.01
There are not opportunities for me to be active	3.54 (0.08)	3.36 (0.06)	−0.09	0.07
In my day to day, there are spaces, situations, or people that encourage me to move more	4.16 (0.07)	4.26 (0.06)	0.05	0.25
**Social norms** Where I work/live, I often see other people being active	5.58 (0.06)	5.57 (0.05)	−0.01	0.94
I know a lot of people who are not active	5.47 (0.06)	5.43 (0.05)	−0.02	0.59
There are more people who use public transport or bicycles compared to cars	3.97 (0.07)	4.06 (0.05)	0.05	0.30
There are more and more people being active	5.75 (0.04)	5.70 (0.04)	−0.04	0.36
***Motivation***				
**Outcome expectancies**				
Physical activity improves my quality of life	6.81 (0.03)	6.88 (0.03)	0.08	0.12
**Interest** Physical activity is as important as other things in my life	5.17 (0.06)	5.46 (0.05)	0.18	<0.001
**Enjoyment**				
I like to do physical activity	5.29 (0.06)	5.48 (0.05)	0.12	0.02
**Locus of causality**				
I do physical activity because I want to, not because I have to	4.60 (0.07)	4.84 (0.06)	0.12	<0.01
**Intention** I intend to do more physical activity in the near future	5.37 (0.06)	5.31 (0.05)	−0.04	0.46

Note: Analyses adjusted for age, sex, education, depression, anxiety, nil diseases and other diseases. Response ranged from 1 (fully disagree) to 7 (fully agree).

**Table 4:** Adjusted physical activity for all respondent’s pre-campaign and post-campaign.

	Pre-Campaign	Post-Campaign	Effect Size (Cohen’s *d*)	*p*-Value
Weekly MET minutes Mean (SE)				
Vigorous	1497 (75)	1769 (61)	0.13	<0.01
Moderate	1164 (48)	1171 (39)	0.01	0.92
Walking	1062 (43)	1102 (35)	0.03	0.47
Total activity	3613 (119)	3921 (98)	0.10	0.05
Sitting	2268 (40)	2222 (32)	−0.04	0.37

Note: Analyses adjusted for age, sex, education, depression, anxiety, nil diseases and other diseases. MET-minutes are the weekly time x the energy expenditure value (METs) assigned to each physical activity.

**Table 5:** Adjusted odds of lifestyle physical activity behaviours post-campaign compared to pre-campaign.

	Pre-Campaign *n* (%)	Post-Campaign *n*(%)	Post-Campaign Compared to Pre-Campaign OR (95% CI)	Effect Size (Cohen’s *h*)	*p*-Value
**In the last month have you often**...					
...climbed the stairs rather than taking the lift	488 (63.3)	766 (66.8)	1.14 (0.94, 1.39)	0.07	0.18
...walked rather than going by car	384 (49.8)	579 (50.5)	1.07 (0.89, 1.3)	0.01	0.45
...parked the car further or got off public transport early to walk more	270 (35)	381 (33.3)	0.97 (0.8, 1.18)	−0.04	0.77
...taken break during work to walk or move more	231 (30)	351 (30.6)	1.08 (0.88, 1.32)	0.01	0.48
...chosen to stand when you could sit	286 (37.1)	438 (38.2)	1.06 (0.88, 1.29)	0.02	0.53
...chosen to do things manually, when you could use machines	223 (28.9)	336 (29.3)	1.08 (0.88, 1.33)	0.01	0.46

Note. Analyses adjusted for age, sex, education, depression, anxiety, nil diseases and other diseases.

## Supplementary Materials

The following are available online at https://www.mdpi.com/1660-4601/17/21/8062/s1, Table S1: *Unadjusted* physical activity for all respondents pre-campaign and post-campaign, Table S2: Adjusted physical activity for all respondents post-campaign by awareness, Table S3: Adjusted lifestyle physical activity behaviours variables for all respondents by awareness (prompted), Table S4: Adjusted theoretical (COM-B) variables for all respondents by awareness (prompted), Table S5: Adjusted theoretical (Com-B) variables for all respondents by awareness (image recognition).
