# Supplementary material for: “Follow the Whistle: Physical Activity Is Calling You”: Evaluation of Implementation and Impact of a Portuguese Nationwide Mass Media Campaign to Promote Physical Activity
Source: Int J Environ Res Public Health. 2020 Nov 2;17(21):8062. doi: 10.3390/ijerph17218062 (PMC7663013; doi:10.3390/ijerph17218062)
Supplement: Supplementary file 1 [file ijerph-17-08062-s001.pdf]

Online supplementary material

Table S1. *Unadjusted* physical activity for all respondents pre-campaign and post-campaign.

	Pre-campaign	Post-campaign	p-value
Weekly MET minutes Mean (SE)			
Vigorous	1,492 (75)	1,772 (62)	<0.01
Moderate	1,181 (48)	1,160 (40)	0.74
Walking	1,080 (43)	1,090 (35)	0.86
Total activity	3,641 (120)	3,902 (98)	0.09
Sitting	2,252 (40)	2,232 (33)	0.70

Table S2. Adjusted physical activity for all respondents post-campaign by awareness.

	Unaware	Aware	p-value
Identified at least one correct campaign image			
Adjusted mean (SE)			
Vigorous	1,798 (101)	1,757 (76)	0.74
Moderate	1,217 (62)	1,127 (47)	0.25
Walking	1,156 (58)	1,052 (44)	0.15
Total activity	4,052 (159)	3,816 (119)	0.23
Sitting	2,167 (54)	2,271 (41)	0.13
Prompted recognition of campaign			
Adjusted mean (SE)			
Vigorous	1,838 (73)	1,580 (119)	0.07
Moderate	1,160 (46)	1,154 (74)	0.95
Walking	1,075 (42)	1,136 (68)	0.44
Total activity	3,955 (116)	3,750 (188)	0.36
Sitting	2,252 (40)	2,213 (63)	0.60

Note: Analyses adjusted for age, sex and education.

Table S3. Adjusted lifestyle physical activity behaviours variables for all respondents by awareness (prompted).

	Var	Unaware	Aware	p-value
In the last month have you...				
...climbed the stairs rather than taking the escalator/elevator	Q31_1			0.70
Not often		Ref	Ref	
Often		1.95 (1.75, 2.17)	1.85 (1.46, 2.35)	
...walked rather than going by car	Q31_2			0.62
Not often		Ref	Ref	
Often		1.03 (0.93, 1.14)	0.96 (0.77, 1.21)	
...parked the car further or got off public transport early to walk more	Q31_3			0.58
Not often		Ref	Ref	
Often		0.51 (0.46, 0.57)	0.47 (0.37, 0.6)	
...taken break during work to walk or move more	Q31_4			0.59
Not often		Ref	Ref	
Often		0.41 (0.37, 0.46)	0.44 (0.35, 0.57)	
...chosen to stand when you could sit	Q31_5			0.77
Not often		Ref	Ref	
Often		0.6 (0.54, 0.66)	0.62 (0.49, 0.78)	
...chosen to do things manually, when you could use machines	Q31_6			
Not often		Ref	Ref	0.64
Often		0.39 (0.35, 0.44)	0.42 (0.32, 0.54)	

Note: Analyses adjusted for age, sex and education.

Table S4. Adjusted theoretical (COM-B) variables for all respondents by awareness (prompted).

	Var	Unaware	Aware	p-value
Perceived capability				
Knowledge				
Climbing stairs or walking are not physical activity	Q20_1	2.26 (0.05)	2.05 (0.11)	0.10
Only high intensity activity has benefits	Q20_2	1.59 (0.03)	1.55 (0.07)	0.64
Self-efficacy				
I am confident that I can keep active on a regular basis, even when....				
I have little time	Q28_1	4.40 (0.05)	4.78 (0.1)	<0.01
I'm not in great physical shape	Q28_2	4.67 (0.04)	4.92 (0.1)	0.02
I do not have specific equipment	Q28_3	4.89 (0.05)	5.20 (0.11)	<0.01
I do not have much money	Q28_4	5.02 (0.05)	5.03 (0.11)	0.92
I can integrate physical activity into my day	Q20_4	5.08 (0.04)	5.78 (0.10)	<0.001
Currently, it is relatively easy for me to walk or bicycle for at least part of my daily journeys	Q26_5	4.18 (0.06)	4.37 (0.13)	0.16
Perceived opportunity				
Physical and social environment				
There are many opportunities to be active where I live	Q26_2	5.24 (0.04)	5.57 (0.1)	<0.001
There are not opportunities for me to be active	Q26_3	3.49 (0.05)	3.12 (0.12)	<0.01
In my day to day life, there are spaces, situations, or people that encourage me to move more	Q26_4	4.16 (0.05)	4.41 (0.11)	0.03
Social norms				
Where I work/live, I often see other people being active	Q29_4	5.55 (0.04)	5.63 (0.10)	0.49
I know a lot of people who are not active	Q30_1	5.46 (0.04)	5.38 (0.09)	0.45
There are more people who use public transport or bicycles compared to cars	Q30_2	3.98 (0.05)	4.06 (0.11)	0.51
There are more and more people being active	Q30_4	5.71 (0.03)	5.74 (0.07)	0.73
Motivation				
Outcome expectancies<br>Physical activity improves my quality of life	Q20_3	6.84 (0.02)	6.96 (0.05)	0.03
Interest<br>Physical activity is as important as other things in my life	Q29_1	5.30 (0.04)	5.52 (0.10)	0.04
Enjoyment<br>I like to do physical activity	Q29_2	5.34 (0.05)	5.63 (0.10)	0.02
Locus of causality<br>I do physical activity because I want to, not because I have to	Q29_3	4.69 (0.05)	4.85 (0.12)	0.23
Intention<br>I intend to do more physical activity in the near future	Q30_3	5.3 (0.04)	5.47 (0.10)	0.09

Note: Analyses adjusted for age, sex and education. Response ranged from 1 (fully disagree) to 7 (fully agree).

Table S5. Adjusted theoretical (Com-B) variables for all respondents by awareness (image recognition).

	Var	Unaware	Aware	p-value
Perceived capability				
Knowledge				
Climbing stairs or walking are not physical activity	Q20_1	2.29 (0.05)	2.08 (0.1)	0.06
Only high intensity activity has benefits	Q20_2	1.6 (0.03)	1.52 (0.06)	0.23
Self-efficacy				
I am confident that I can keep active on a regular basis, even when....				
I have little time	Q28_1	4.41 (0.05)	4.73 (0.09)	<0.01
I'm not in great physical shape	Q28_2	4.67 (0.04)	4.90 (0.08)	0.02
I do not have specific equipment	Q28_3	4.92 (0.05)	5.00 (0.1)	0.48
I do not have much money	Q28_4	5.00 (0.05)	5.03 (0.1)	0.83
I can integrate physical activity into my day	Q20_4	5.06 (0.04)	5.71 (0.09)	<0.001
Currently, it is relatively easy for me to walk or bicycle for at least part of my daily journeys	Q26_5	4.17 (0.06)	4.42 (0.11)	0.04
Perceived opportunity				
Physical and social environment				
There are many opportunities to be active where I live	Q26_2	5.25 (0.04)	5.53 (0.08)	<0.01
There are not opportunities for me to be active	Q26_3	3.49 (0.05)	3.21 (0.1)	0.02
In my day to day life, there are spaces, situations, or people that encourage me to move more	Q26_4	4.16 (0.05)	4.45 (0.09)	<0.01
Social norms				
Where I work/live, I often see other people being active	Q29_4	5.54 (0.04)	5.71 (0.08)	0.07
I know a lot of people who are not active	Q30_1	5.45 (0.04)	5.41 (0.08)	0.66
There are more people who use public transport or bicycles compared to cars	Q30_2	3.97 (0.05)	4.22 (0.09)	<0.01
There are more and more people being active	Q30_4	5.69 (0.03)	5.82 (0.06)	0.05
Motivation				
Outcome expectancies				
Physical activity improves my quality of life	Q20_3	6.82 (0.02)	6.94 (0.04)	0.02
Interest				
Physical activity is as important as other things in my life	Q29_1	5.31 (0.04)	5.48 (0.08)	0.07
Enjoyment				
I like to do physical activity	Q29_2	5.36 (0.05)	5.56 (0.09)	0.04
Locus of causality				
I do physical activity because I want to, not because I have to	Q29_3	4.73 (0.05)	4.81 (0.1)	0.43
Intention				
I intend to do more physical activity in the near future	Q30_3	5.32 (0.04)	5.39 (0.08)	0.43

Note: Analyses adjusted for age, sex and education. Response ranged from 1 (fully disagree) to 7 (fully agree).
